# Mindfulness skills and experiential avoidance as therapeutic mechanisms for treatment-resistant depression through mindfulness-based cognitive therapy and lifestyle modification

**DOI:** 10.3389/fpsyg.2023.1008891

**Published:** 2023-03-09

**Authors:** Mauro Garcia-Toro, Alejandra Aguilar-Latorre, Aurora Garcia, Capilla Navarro-Guzmán, Elena Gervilla, Andrea Seguí, Francisco Gazquez, Jose Antonio Marino, Rocío Gomez-Juanes, María J. Serrano-Ripoll, Bárbara Oliván-Blázquez, Javier Garcia-Campayo, Shannon Maloney, Jesús Montero-Marin

**Affiliations:** ^1^University Institute of Health Science Research (IUNICS), University of the Balearic Islands, Palma, Spain; ^2^Health Research Institute of the Balearic Islands (IdISBa), Palma, Spain; ^3^Department of Medicine, University of the Balearic Islands, Palma, Spain; ^4^Research Network on Chronicity, Primary Care and Health Promotion (RICAPPS), Carlos III Health Institute, Madrid, Spain; ^5^Institute for Health Research Aragón (IIS Aragón), Zaragoza, Spain; ^6^Department of Psychology, University of the Balearic Islands, Palma, Spain; ^7^Primary Care Research Unit of Mallorca, Balearic Islands Health Services, Palma, Spain; ^8^Department of Psychology and Sociology, University of Zaragoza, Zaragoza, Spain; ^9^Department of Medicine, Psychiatry and Dermatology, University of Zaragoza, Zaragoza, Spain; ^10^Department of Psychiatry, Warneford Hospital, University of Oxford, Oxford, United Kingdom; ^11^Teaching, Research and Innovation Unit, Parc Sanitari Sant Joan de Déu, Sant Boi de Llobregat, Spain; ^12^Consortium for Biomedical Research in Epidemiology and Public Health (CIBER Epidemiology and Public Health-CIBERESP), Madrid, Spain

**Keywords:** major depressive disorder, MBCT, lifestyle modification, mediation, intervention

## Abstract

**Background/objective:**

The COVID-19 pandemic and consequent physical distancing has made it difficult to provide care for those with Treatment-Resistant Depression (TRD). As a secondary analysis of a clinical trial, the aim of this study was to explore potential mechanisms through which three online-delivered approaches, added to treatment as usual, improve depressive symptoms in TRD patients.

**Methods:**

The three approaches included (a) Minimal Lifestyle Intervention (MLI), (b) Mindfulness-Based Cognitive Therapy (MBCT), and (c) Lifestyle Modification Program (LMP). Sixty-six participants with TRD completed assessments pre-post intervention (mindfulness skills [FFMQ]; self-compassion [SCS]; and experiential avoidance [AAQ-II]) and pre-intervention to follow-up (depressive symptoms [BDI-II]). Data were analyzed using within-subjects regression models to test mediation.

**Results:**

Mindfulness skills mediated the effect of MBCT on depressive symptoms (*ab* = −4.69, 95% CI = −12.93 to−0.32), whereas the lack of experiential avoidance mediated the effect of LMP on depressive symptoms (*ab* = −3.22, 95% CI = −7.03 to−0.14).

**Conclusion:**

Strengthening mindfulness skills and decreasing experiential avoidance may promote recovery in patients with TRD, MBCT, and LMP have demonstrated that they may help increase mindfulness skills and decrease experiential avoidance, respectively. Future work will need to unpick the components of these interventions to help isolate active ingredients and increase optimization.

## Introduction

Treatment-resistant depression (TRD) is identified when individuals with major depressive disorder (MDD) have not responded to at least two different antidepressant treatments at the time of a moderate to severe depressive episode. It has been estimated that 50–60% of those with MDD are treatment-resistant and the prevalence of MDD is increasing along with its associated costs (e.g., low quality of life, absenteeism, healthcare visits, etc.,) (Fava, 2003; Mrazek et al., 2014). The first-line treatments for MDD are pharmacotherapy and psychotherapy. However, there are voices proposing that efforts should be directed at investigating integrative or adjunctive interventions, which combine these first-line treatments with other complementary ones, to enhance outcomes for depression (Lopresti, 2019; Foroughi et al., 2020; Newland and Bettencourt, 2020).

Psychotherapies, such as cognitive behavioral therapy (CBT), have demonstrated efficacy in TRD, especially as an integrative approach with antidepressant medications (Carvalho et al., 2014; Ijaz et al., 2018). Given the evidence for mindfulness-based cognitive therapy (MBCT) in the context of recurrent depression in reducing residual depressive symptoms and depressive relapse (Kuyken et al., 2016; McCartney et al., 2021), and its theoretical overlap with CBT, there is a rationale for exploring its efficacy in the context of TRD. Other approaches such as lifestyle modifications (e.g., physical exercise, and diet) have been recommended since one habit change tends to lead to other positive changes and, alongside antidepressant medications, may be an appropriate integrative approach to addressing depressive symptoms in TRD (Al-Harbi, 2012; Navarro et al., 2020). Although there is preliminary evidence for both MBCT and lifestyle modification in the context of TRD (García-Toro et al., 2012; Eisendrath et al., 2016; Cladder-Micus et al., 2018; ter Avest et al., 2019; Foroughi et al., 2020), more trials are needed to understand the specific effects of these approaches and their usefulness in the medium-to-long term (Wang et al., 2018; Wong et al., 2021). There is limited to no research on the relative efficacy of these approaches in the context of TRD.

In addition to investigating efficacy, investigating the mechanisms of effective psychosocial therapeutic interventions for TRD is also crucial to improve our understanding of active components and their relative importance (Gu et al., 2015; Pérez-Aranda et al., 2021). With an increased understanding, the most determining components can be enhanced and consequently the effectiveness of these programs can be strengthened and optimized (van der Velden et al., 2015; Alsubaie et al., 2017). Several mechanisms in the context of MBCT have been proposed and three overlapping yet distinct factors have received considerable consensus: mindfulness skills, self-compassion, and (reduced) experiential avoidance (Gu et al., 2015; Collado-Navarro et al., 2021; Medlicott et al., 2021; Yela et al., 2022). Mindfulness has been defined as awareness that arises through purposefully paying attention to the present moment and doing so without judgment (Cebolla et al., 2012; Parmentier et al., 2019). Self-compassion has been defined as being kind to oneself when confronting personal inadequacies or situational difficulties, framing the imperfection of life in terms of common humanity, and being mindful of negative emotions so that one neither suppresses nor ruminates on them (Carona et al., 2022; Neff and Germer, 2022). Experiential avoidance has been defined as the unwillingness to remain in contact with difficult private experiences such as painful thoughts and emotions (Chawla and North, 2007; Aguilera et al., 2019).

In the context of lifestyle modification interventions, mechanisms of action in relation to depressive symptoms have not been established yet, but it is proposed that they could be related to the promotion of social behavior, from a psychological point of view, and anti-inflammatory and antioxidant properties at the biological level (Hidaka, 2012; Gómez-Donoso et al., 2020). Ultimately, to understand the extent to which change in the proposed mechanisms (i.e., mindfulness skills, self-compassion, and experiential avoidance) are specific to MBCT, the current study examined these constructs across three psychotherapeutic approaches: (a) Minimal Lifestyle Intervention (MLI), (b) Mindfulness-Based Cognitive Therapy (MBCT), and (c) Lifestyle Modification Program (LMP). Overall, past research examining potential mechanisms of mindfulness-based programs are largely limited to methodologically flawed designs (Gu et al., 2015). For example, there are few studies that have evaluated proposed mechanisms longitudinally (e.g., before, during, and after delivering the intervention) (Yela et al., 2022). Additionally, there are few studies that have investigated clinical MDD populations with TRD and, as a result, there is only preliminary and insufficient evidence evaluating mechanisms at this time. (Gu et al., 2015; van der Velden et al., 2015; Parmentier et al., 2019; Medlicott et al., 2021; Pérez-Aranda et al., 2021).

The current study aims to address the relative efficacy of three psychotherapeutic approaches in the context of TRD by exploring the specificity of three potential mechanisms (mindfulness skills, self-compassion, and experiential avoidance). In light of the COVID-19 pandemic and the subsequent heightened symptoms (e.g., increased suicidality) in many individuals with clinically stable MDD (Zhang et al., 2022), and the difficulties to speak to a healthcare professional face-to-face, we examined these three psychotherapeutic approaches using an online format in an effort to find a solution for these individuals. Due to the exploratory nature of the study, the aim is to ultimately generate hypotheses regarding the potential mechanisms of these three approaches (MLI, MBCT, and LMP) in relation to depressive symptoms in TRD.

## Methods

This study was a secondary analysis of a pragmatic parallel randomized controlled clinical trial that was composed of three arms (MLI, MBCT, and LMP). The parent paper (Garcia et al., 2022) addresses the primary aim of examining the relative efficacy of these approaches in reducing depressive symptoms at 12-month follow-up. The current paper is a secondary analysis that evaluates potential mechanisms of change. Due to the limited understanding of medium to long term effects, and the issue with temporal precedence with examining mechanisms of change, the current study will include a 6-month follow-up time-point and will measure potential mechanisms before and after the intervention. Details of the main study methods and procedures are published in the corresponding protocol (Navarro et al., 2020).The trial was registered with ClinicalTrials.gov (NCT04428099; 11-06-2020).

### Participants

We recruited individuals from the Primary Healthcare Centers of the Balearic Islands region in Spain who were currently experiencing an episode of TRD. The inclusion criteria were: (Fava, 2003) 18 years old or older; (Mrazek et al., 2014) having a diagnosis of MDD based on the DSM-5 and Mini-International Neuropsychiatric Interview (MINI) criteria (Ferrando et al., 2000); (Foroughi et al., 2020) having at least two failed antidepressant treatment attempts or refusal of antidepressant treatment during the current episode; (Lopresti, 2019) having experienced at least 1 month of treatment with a psychiatrist and/or psychologist during the current episode; (Newland and Bettencourt, 2020) being able to understand written and spoken Spanish; (Carvalho et al., 2014) being able to provide written informed consent; and (Ijaz et al., 2018) having knowledge of and access to technologies to engage in online video conferences.

The exclusion criteria were: (Fava, 2003) the presence of another condition that affects the central nervous system (e.g., organic brain pathology, traumatic brain injury, or dementia); (Mrazek et al., 2014) having another psychiatric diagnosis (e.g., substance dependence or abuse, history of schizophrenia or other psychotic disorders, eating disorders) except for anxiety or personality disorders; (Foroughi et al., 2020) the presence of a medical, infectious, or degenerative condition that may interfere with affective symptoms; (Lopresti, 2019) the presence of delirium or hallucinations; and (Newland and Bettencourt, 2020) a relevant risk of suicide.

A total of 94 patients were randomized to MLI (*n* = 31), MBCT (*n* = 29), or LMP (*n* = 34), and were assessed at baseline. In the MLI group, 18 participants completed assessments at post-test and at follow-up, and thus, 13 participants had missing data in the MLI group. In the MBCT group, 22 participants completed assessments at post-test and at follow-up, and thus, 7 participants had missing data in the MBCT group. In the LMP group, 26 participants completed assessments at post-test and at follow-up, and thus, 8 participants had missing data in the LMP group. Finally, 66 patients who completed all the assessment surveys (i.e., complete cases) were included in the present study. The socio-demographic characteristics of participants are shown in Table 1.

**Table 1:** Demographic characteristics of participants at baseline.

Variables	MLI (*n* = 18)	MBCT (*n* = 22)	LMP (*n* = 26)	*p*
Age, *M* (*SD*)	43.89 (12.40)	52.82 (13.87)	46.96 (13.69)	0.943
Gender, female, *n (%)*	13 (72.0)	19 (86.4)	17 (65.4)	0.274
*Education*				
Primary or less, n (%)	3 (16.7)	3 (13.6)	6 (23.1)	0.724
Secondary or more, n (%)	15 (83.3)	19 (86.4)	20 (76.9)	
*Occupation*				
Working, n (%)	6 (33.3)	4 (18.2)	8 (30.8)	0.541
Not working, n (%)	12 (66.7)	18 (81.8)	18 (69.2)	
*Marital status*				
With a partner, n (%)	5 (27.8)	4 (18.2)	10 (38.5)	0.299
Without a partner, n (%)	13 (72.2)	18 (81.8)	16 (61.5)	
Taking antidepressants, yes n (%)	13 (72.2)	16 (72.7)	24 (92.3)	0.134

*p*: value of *p* for the one-way ANOVA or Fisher’s exact test. MLI, minimal lifestyle intervention; MBCT, mindfulness-based cognitive therapy; LMP, lifestyle modification program.

### Procedure and ethics

Individuals from the Balearic Islands in Spain, who were currently experiencing a TRD episode, were recruited by cooperating with mental health workers and submitting applications online. Specifically, the information about the study was disseminated by the members of the research team through social networks and their professional contacts (e.g., associations and patient groups, and groups of mental health professionals). To ensure that the participants met the inclusion criteria, a trained psychologist administered the Mini-International Neuropsychiatric Interview (MINI) (Ferrando et al., 2000) by telephone. If a participant was eligible, the psychologist administered the baseline survey. After agreeing to participate and once their informed consent had been provided, eligible participants were randomly assigned to MLI, MBCT, or LMP in a 1:1:1 ratio. Randomization was performed using a computer-generated random assignment sequence with random Allocation Software (v.2.0) (Saghaei, 2004). An independent researcher from a hospital unit, not involved in the clinical trial, performed the randomization. The three interventions were carried out remotely and delivered online. Two therapists ran each psychotherapeutic intervention: a psychologist certified in MBCT and a mental health nurse with previous experience in the use of lifestyle programs in patients with depression. Both led the four groups of 12–18 participants that were formed on a rolling-basis over the course of 18 months that the recruitment lasted. Two intervention groups were performed for each treatment arm. Participants completed the battery of questionnaires, alongside the psychologists, by telephone.

#### Minimal lifestyle intervention

The MLI group received, in addition to treatment as usual (TAU), individual online messages with basic written suggestions for lifestyle changes once after agreeing to take part in the study. These recommendations were aimed at improving their depressive symptoms by suggesting going to bed early, practice good sleep habits, walk at least 1 hour a day, expose themselves to the sun for 2 hours a day, eat a healthy and balanced diet, and try to improve their social support network (Serrano-Ripoll et al., 2015). Across all three interventions, TAU included antidepressants and/or psychotherapy depending on individual preference.

#### Mindfulness-based cognitive therapy

The MBCT group received, in addition to TAU, the original eight-week MBCT for depression protocol developed by Segal et al. (2013). This protocol, adapted for an online format, included 8 weeks of weekly group-based sessions (3 h per session). These weekly online sessions covered the following topics: the causes, symptoms, and course of depression; treatment for depression; attention; recognizing “automatic pilot”; tuning into the breath and body; relating to direct experience; labeling experiences; being fully present in the moment; approaching daily life with curiosity and openness; letting things be as they are; recognizing thoughts and feelings as mental processes that may not correspond with reality; caring for oneself and with self-compassion; and bringing mindfulness into one’s daily life (Segal et al., 2013).

#### Lifestyle modification program

The LMP group received, in addition to TAU, eight weeks of weekly group-based sessions (3 h per session). These weekly sessions covered the following topics: the causes, symptoms, and course of depression; treatment for depression; the importance of physical exercise for physical and mental health; how to exercise safely and comfortably; how to be motivated to incorporate physical exercise into daily routines; the importance of good nutrition, a social support network, and appropriate sleep–wake rhythms and sleep hygiene; the role of negative rumination in maintaining depression along with strategies to detect and stop this behavior; and the importance of getting outside and connecting with nature. Practical recommendations were made on how to combine some of these suggestions for long-term use (Aguilar-Latorre et al., 2020; Navarro et al., 2020; Aguilar-Latorre et al., 2022).

Ethics approval was granted by the Research Ethics Committee of the Balearic Islands (IB3925/19PI; 29-05-2019). The study was developed in accordance with the Helsinki Declaration and its updates. All subjects provided informed consent before group allocation. All collected data was processed as stipulated in the current Spanish legislation regarding the protection of personal data (Law 3/18, Dec 5th). Once the data was collected it was anonymized for data analysis and only used for the study purposes.

### Measurements

We collected sociodemographic information at baseline on age, gender (“female,” or “male”), education (“primary or less,” including people without studies, with incomplete primary studies, and with complete primary studies; and “secondary or more,” including people with complete secondary studies and/or complete university studies), occupation (“working,” or “not working”), and marital status (“with,” or “without” a partner).

The primary outcome was self-reported symptoms of depression using the Spanish version of the Beck Depression Inventory-II (Sanz et al., 2005), and it was measured at pre-intervention and at the 6-month follow-up. This 21-item questionnaire measures the severity of depressive symptoms and is responded to on a scale of 0 to 3 (e.g., “*I do not feel sad”* to *“I am so sad or unhappy that I cannot stand it”*), giving a total score that ranges between 0 and 63. Higher scores are associated with greater symptom severity for depression. The cut-offs are: 0–13 (minimal depression); 14–19 (mild depression); 20–28 (moderate depression); and 29–63 (severe depression). The internal consistency of the BDI-II in our sample at baseline was appropriate, with a Cronbach’s *α* = 0.85.

Mechanistic outcomes include self-reported mindfulness skills, self-compassion, and experiential avoidance. Mindfulness skills were assessed using the Spanish version of the Five Facet Mindfulness Questionnaire (FFMQ; (Cebolla et al., 2012)), measured pre-post intervention. This 39-item questionnaire assesses: the observation of experiences such as sensations, thoughts, or emotions; description of internal experiences; awareness of actions; non-judgmental stances toward thoughts and feelings; and non-reactivity toward thoughts and feelings (Cebolla et al., 2012). This questionnaire is responded to on a scale of 1 to 5 (*“never or very rarely true”* to *“very often or always true”*). The total score is the sum of the direct and reverse-scored items and ranges between 39 and 195, with higher scores associated with higher levels of mindfulness. The internal consistency of the FFMQ in our sample at pre-intervention showed adequate values, with *α* = 0.85.

Self-compassion was assessed using the Spanish version of the Self-Compassion Scale-Short Form (SCS-SF; (Garcia-Campayo et al., 2014), measured pre-post intervention. This 12-item questionnaire assesses how respondents perceive their actions toward themselves during difficult times and measures the following six components of self-compassion (with reverse coding of negative aspects): self-kindness, self-judgment, common humanity, isolation, mindfulness, and over-identification. Each item is rated on a scale of 1 to 5 (*“almost never”* to *“almost always”*). The total score is the sum of the direct and reverse-scored items and ranges between 12 and 60, with higher scores associated with greater levels of self-compassion. The internal consistency of the SCS-SF in our sample at pre-intervention was good, with *α* = 0.75.

Experiential avoidance was assessed using the Spanish version of the Acceptance and Action Questionnaire-II (AAQ-II; (Ruiz et al., 2013), measured pre-post intervention. This 7-item questionnaire assesses the unwillingness to experience unwanted emotions and thoughts, and the inability to be in the present moment and commit to value-directed actions. Each item is responded to on a scale of 1 to 7 (*“never true”* to *“always true”*). The total score ranges from 7 to 49, with higher scores associated with greater levels of experiential avoidance. The internal consistency of the AAQ-II at baseline was *α* = 0.83.

### Statistical analyzes

Descriptive analysis (frequencies and percentages for categorical variables; means and standard deviations (SDs) for continuous variables), and a univariate analysis (one-way ANOVA and Fisher’s exact probability test) were used to examine between-group differences in sociodemographic data across the three interventions at baseline.

Within-group t-tests were used to evaluate the pre-post changes in mindfulness skills, self-compassion, and experiential avoidance (i.e., potential mediators), and the pre-intervention to follow-up changes in depressive symptoms (i.e., primary outcome). We calculated Hedges’ *g* as an effect size measure, which corrects for potential small sample bias, for each within-group comparison. Hedges’ *g* is considered small when *g* = 0.20, moderate when *g* = 0.50, and large when *g* ≥ 0.80 (Cohen, 1988). Between-group ANCOVAs at post-intervention (for potential mediators), and follow-up (for primary outcome) were conducted, with the baseline assessment acting as a covariate, and partial eta-squared (*ƞ^2^*) as the effect size measure [*ƞ^2^* ≤ 0.01 is small, *ƞ^2^* = 0.06 is medium, and *ƞ^2^* ≥ 0.14 is large] (Cohen, 1988). Descriptive statistics (means, and SDs) were also calculated.

The potential mediating effects of mindfulness skills, self-compassion and experiential avoidance on depressive symptoms were evaluated using a within-subjects design by regressing the change in depressive symptoms (pre-intervention to follow-up) on the change in each potential mediator (pre-post intervention) using ordinary least squares (OLS) regression models for each intervention group independently. This analytical approach involves a test of mediation in which the same individuals are measured at distinct time-points and includes a design in which the factor that causes the dependence is crossed with the independent variable, which is the repeated-measures factor. Mediation is present when the difference in the dependent variable depends on the difference in the mediator (Judd et al., 2001). Using a path analysis framework, we explored the indirect relationships between the repeated-measures factor, the mediator pre-post difference, and the depressive symptoms pre-intervention to follow-up difference, using an OLS analysis with unstandardized path estimates from regression coefficients whereby: (i) the repeated-measures factor is the independent variable (*X*), (ii) the mediator pre-post difference is the mediating factor (M), and (iii) the depressive symptoms pre-intervention to follow-up difference is the dependent variable (*Y*). The regression coefficient and its 95% confidence interval (95% CI) for the bootstrapped (5,000 samples) indirect effects were calculated. This test is applied to overcome potential problems of asymmetry in the distribution of the indirect effects (Lockhart et al., 2011), which are statistically significant when the 95% CI of the “*ab*” parameter does not include zero. Multiple determination coefficients (*R^2^*) were calculated to observe the explanatory power of the mediating models.

The significance level was set at 0.05 using two-sided tests. Given the exploratory nature of this study, we did not correct for multiple testing. The analyzes followed a complete case approach using SPSS v27.0 software (IBM Corp, 2017).

## Results

Table 1 summarizes the main demographic characteristics of our sample at baseline, comparing the three study arms. Most participants were middle-aged women; more than three-quarters of the sample had completed secondary or university education; and around two-thirds were not working and had no partner. No differences were found across the three arms in terms of baseline demographic characteristics.

As shown in Table 2, significant pre-intervention to follow-up within-group improvements were observed in all the groups for depressive symptoms, with large effects. In addition, significant pre-post within-group improvements were also observed in all the groups for mindfulness skills, self-compassion, and experiential avoidance with moderate to large effects, except for self-compassion within the LMP group, which demonstrated a small non-significant effect. There were no significant between-group differences at follow-up for depressive symptoms after controlling for baseline levels (*F* = 0.58; *p* = 0.563; *ƞ^2^* = 0.02), and at post-intervention for mindfulness skills (*F* = 0.44; *p* = 0.644; *ƞ^2^* = 0.02), self-compassion (*F* = 2.03; *p* = 0.139; *ƞ^2^* = 0.06) and experiential avoidance (*F* = 0.79; *p* = 0.460; *ƞ^2^* = 0.02) after controlling for baseline levels.

**Table 2:** Within-group analyzes of depressive symptoms and potential mediators.

Outcomes/time points	MLI	*g*	MBCT	*g*	LMP	*g*
*Depressive symptoms*						
Baseline	39.11 (12.29)		36.96 (13.29)		33.73 (12.36)	
Follow-up	25.00 (18.70)	0.71^**^	20.55 (11.49)	1.17^***^	17.96 (14.38)	1.22^***^
*Mindfulness skills*						
Baseline	106.79 (17.04)		110.10 (21.50)		103.65 (20.11)	
Post-intervention	116.05 (22.81)	0.41^*^	124.70 (32.23)	0.58^**^	115.52 (22.01)	0.47^*^
*Self-compassion*						
Baseline	24.05 (6.95)		27.09 (6.89)		28.35 (8.04)	
Post-intervention	29.91 (10.03)	0.74^**^	33.91 (9.34)	0.94^***^	30.28 (7.71)	0.21
*Experiential avoidance*						
Baseline	38.50 (7.75)		34.13 (10.74)		38.67 (7.33)	
Post-intervention	33.64 (10.11)	0.43^*^	29.35 (10.17)	0.51^*^	34.11 (7.87)	0.43^*^

Descriptive statistics: means and SDs. g: Hedges’ g (within-group analysis). MLI, minimal lifestyle intervention; MBCT, mindfulness-based cognitive therapy; LMP, lifestyle modification program. ***p < 0.001; **p < 0.01; *p < 0.05.

In the MBCT group, participants at post-intervention showed improvements in mindfulness skills (*a* = 14.47; *p* = 0.023) which predicted an improvement in depressive symptoms at follow-up (*b* = −0.32; *p* = 0.021). A bias-corrected bootstrap confidence interval for the indirect effect (*ab* = −4.69) based on 5,000 bootstrap samples did not include zero (95% CI = −12.93 to-0.32). However, there was evidence that the repeated-measures factor associated with the intervention influenced the change in depressive symptoms independent of its effects on mindfulness skills (*c* = −13.47; *p* < 0.001). Furthermore, in the LMP group, participants at post-intervention showed reductions in experiential avoidance (*a* = −3.96; *p* = 0.032), which predicted an improvement in depressive symptoms at follow-up (*b* = 0.81; *p* = 0.001). A bias-corrected bootstrap confidence interval for the indirect effect (*ab* = −3.22) based on 5,000 bootstrap samples did not include zero (95% CI = −7.03 to-0.14). However, there was evidence that the repeated-measures factor associated with the intervention influenced the change in depressive symptoms independent of its effects on experiential avoidance (*c* = −12.86; *p* < 0.001). No significant indirect effects were observed in the MLI group through mindfulness skills, self-compassion, or experiential avoidance (Table 3).

**Table 3:** Indirect effects of minimal lifestyle intervention, mindfulness-based cognitive therapy, and lifestyle modification program on depressive symptoms through mindfulness skills, self-compassion, and experiential avoidance.

Treatment/Mediators	*R^2^*	*ab*	*SE*	*Boot LLCI*	*Boot ULCI*
*Minimal lifestyle intervention*					
Mindfulness skills	0.09	−2.33	3.51	−12.24	1.48
Self-compassion	0.11	−4.75	4.45	−13.01	5.29
Experiential avoidance	0.12	−1.43	1.91	−5.14	3.04
*MBCT*					
Mindfulness skills	0.30	−4.69	3.48	−12.93	−0.32
Self-compassion	0.01	−0.67	3.39	−6.93	6.60
Experiential avoidance	0.17	−3.12	2.51	−8.54	1.18
*Lifestyle modification program*					
Mindfulness skills	0.25	−2.95	1.91	−6.87	0.44
Self-compassion	0.21	−0.85	1.22	−3.40	1.45
Experiential avoidance	0.40	−3.22	1.82	−7.03	−0.14

*R*^2^: determination coefficient. ab: unstandardized regression coefficient for the indirect effect. SE, bootstrapped standard error. Boot LLCI/ULCI, bootstrapped lower-limits/upper-limits of the 95% confidence interval (based on 5,000 bootstrap samples). MBCT, mindfulness-based cognitive therapy.

## Discussion

The primary aim of this secondary analysis paper was to generate hypotheses regarding the potential mechanisms (e.g., mindfulness skills, self-compassion, and experiential avoidance) of three different teletherapy psychotherapeutic approaches in the context of TRD. The results of this randomized clinical trial demonstrated significant within-group improvements in depressive symptoms at six-month follow-up for all three approaches, with no significant between-group differences. In general, significant within-group improvements were demonstrated in the proposed mechanisms pre-post interventions for all three approaches. However, mindfulness skills only predicted improvements in depressive symptoms within the MBCT arm and experiential avoidance only predicted improvements in depressive symptoms within the LMP arm. Within the MLI arm, none of the proposed mechanisms predicted change in depressive symptoms, which may be due to lack of specificity in terms of the intervention-type. Overall, the findings provide preliminary evidence to suggest that these approaches produce effects through mindfulness skills and experiential avoidance as potential mechanisms of change. Particularly, mindfulness skills might be enhanced by MBCT, which may help reduce reactive responses to unpleasant emotions and reduce rumination (Segal et al., 2013). In LMP, experiential avoidance might be reduced through increased behavioral activation, which is a key feature of this psychotherapy (Collado-Navarro et al., 2021).

These exploratory results suggest that the development of mindfulness skills in MBCT may be important in addressing depressive symptoms in the context of TRD. Several systematic reviews note that mindfulness skills are the most consistent therapeutic mechanism in the context of MBCT, mindfulness-based stress reduction (MBSR), and in individuals with different symptom profiles (Gu et al., 2015; van der Velden et al., 2015; Alsubaie et al., 2017). Moreover, theoretical frameworks of severe clinical depression propose mindfulness as a potential mechanism of change. Thus, our results reinforce this theoretically proposed mechanism in the context of MBCT. In terms of the LMP arm, only experiential avoidance explained the effect on depressive symptoms. This construct has been shown to be closely linked to the severity of depression in such a way that it decreases when depressive symptoms improve, whether this occurs spontaneously or because of the intervention (MacBeth and Gumley, 2012; Gu et al., 2015; Aguilera et al., 2019; Neff and Germer, 2022). Perhaps the personalization of the objectives in the LMP group, which was based on the characteristics of the participants, could have enhanced their exposure to new situations which fostered an empathic, friendly, and mutually supportive environment. This, in turn, could have reduced the participants’ experiential avoidance, acting as a pathway of change for improving their depressive symptoms. It is noteworthy that the MBCT group did not demonstrate this effect. Nevertheless, this is not entirely surprising given that it has not been possible to demonstrate the role of experiential avoidance in a consistent way in the previous literature in the context of MBCT for depression (Gu et al., 2015).

Across all three approaches, self-compassion did not significantly predict change in depressive symptoms. It is possible that the original MBCT protocol (Segal et al., 2013) does not dedicate enough time to working on this construct, which has been noted as being important in other programs for those with depression (Collado-Navarro et al., 2021; Carona et al., 2022; Yela et al., 2022). There are some studies in which self-compassion has operated as a potential mediating factor in the therapeutic change of MBCT, but these studies were only exceptionally applied to treatment-resistant individuals who present a psychopathological severity (e.g., deep ideas of guilt and unworthiness) (van der Velden et al., 2015; Alsubaie et al., 2017; Foroughi et al., 2020). Studies that have investigated the mechanisms for antidepressant therapeutic change in mindfulness-based programs in general have found preliminary but insufficient evidence for the potential mediating role of self-compassion (Gu et al., 2015; Medlicott et al., 2021). In summary, it is critical to focus on the training of specific components that we have seen may facilitate potential improvements (e.g., mindfulness skills in MBCT, and experiential avoidance in LMP) to help maximize benefits.

In terms of limitations, this study was a secondary analysis of a pragmatic randomized clinical trial that aimed to evaluate the adjuvant efficacy of these three approaches to TAU. Based on this design, the secondary analysis conducted cannot fully unpick the extent to which the mechanisms are targeted by TAU, the therapeutic approach, or a combination of the two. Mixed interventions are undoubtedly not ideal when establishing the specific mechanisms of each one but, in this case, it was considered the most appropriate approach for clinical and ethical reasons (van der Velden et al., 2015). The small number of participants in each group and this having implications in terms of statistical power, must be acknowledged and future research will need to investigate these research questions with a larger sample size. In addition, the measurement instruments relied on self-report, which could be biased due to social desirability. Finally, the study was carried out online during the COVID-19 pandemic, which was not initially planned in the original protocol. Face-to-face human contact is an ingredient of the therapeutic relationship that in the online interventions we tried to supply by generating an empathetic climate and group support. However, it is possible that we were not able to achieve this to the same degree as face-to-face delivery. Future studies should evaluate the quality of delivering these interventions and examine our research questions under different circumstances. Nevertheless, the use of an online intervention may also be a strength in a post-pandemic context as it arguably can reach wider audiences, and this opens possible lines of comparative research between interventions carried out face-to-face and online.

The current study has provided support for maintaining intervention programs online for those with TRD. The novelty of this study, having been carried out during unprecedented circumstances (for individuals with TRD, clinicians, and researchers) under a generalized stressor (i.e., the COVID-19 pandemic), is seen as a major strength given that the results suggest viability and potential usefulness. However, the question remains as to whether these mechanisms would have behaved in the same way in less exceptional circumstances, which would have made it possible to carry out the programs in the usual face-to-face in-person format. As we had a homogeneous sample of participants who met the DSM-5 criteria for MDD, as well as a prospective design with different active groups and time-points, we were able to contribute to this field of research in an exploratory and hypotheses-generating way. Past research that has examined the influence of the proposed mechanisms in MLI, MBCT, and LMP have been restricted to cross-sectional or longitudinal designs without active comparators (Gu et al., 2015; Pérez-Aranda et al., 2021). Even the studies which have evaluated these kinds of psychological mechanisms in individuals with MDD have rarely studied the disorder in the acute phase and have tended to only examine potential mechanisms in remitters (van der Velden et al., 2015).

## Conclusion

Based on our exploratory results of this clinical trial, all three approaches (MLI, MBCT, and LMP) demonstrated promising results in improving depressive symptoms in TRD. However, the key mechanisms of action underlying these approaches may differ. For instance, the results of this paper found that mindfulness skills mediated effects in MBCT, whereas the reduction of experiential avoidance mediated effects in LMP. Therefore, this study provides support for the use of multicomponent programs that promote the development of mindfulness skills and reducing experiential avoidance in TRD patients. A special focus on the development of core components underlying these interventions, that may best explain therapeutic benefit in this context (e.g., mindfulness skills in MBCT, and experiential avoidance in LMP), will help optimize the effectiveness of these therapeutic approaches. Examining which other potential mechanisms may be relevant to this population of people with TRD (e.g., group support, therapeutic adherence improvement, etc.,) is also an area of work that warrants further investigation.

## Data availability statement

The raw data supporting the conclusions of this article will be made available by the authors, without undue reservation.

## Ethics statement

The studies involving human participants were reviewed and approved by Research Ethics Committee of the Balearic Islands (IB3925/19PI; 29-05-2019). The patients/participants provided their written informed consent to participate in this study.

## Author contributions

MG-T, AA-L, and JM-M drew up the research design. AG, CN-G, EG, AS, FG, JAM, RG-J, and MS-R have participated in the intervention and helped with project coordination. MG-T is the principal investigator of the project. MG-T, AA-L, SM, and JM-M drafted the protocol and wrote the manuscript. All authors contributed to the article and approved the submitted version.

## Funding

This work was supported by the Spanish Ministry of Science, Innovation and Universities grant number RTI2018-093590-B-I00, research project financed by MCIN/AEI/10.13039/501100011033/ and FEDER FUNDS “Another way to make Europe.”

## Conflict of interest

The authors declare that the research was conducted in the absence of any commercial or financial relationships that could be construed as a potential conflict of interest.

The reviewer JH declared a shared affiliation with the authors AA-L, BO-B, and JG-C to the handling editor at the time of review.

## Publisher’s note

All claims expressed in this article are solely those of the authors and do not necessarily represent those of their affiliated organizations, or those of the publisher, the editors and the reviewers. Any product that may be evaluated in this article, or claim that may be made by its manufacturer, is not guaranteed or endorsed by the publisher.
